# Bioinformatics of genome regulation and structure – 2020 papers collection

**DOI:** 10.1515/jib-2020-0038

**Published:** 2020-12-22

**Authors:** Yuriy L. Orlov, Oxana B. Dobrovolskaya, Ming Chen, Ralf Hofestädt

**Affiliations:** The Digital Health Institute, I.M. Sechenov First Moscow State Medical University (Sechenov University), 119991 Moscow, Russia; Novosibirsk State University, 630090 Novosibirsk, Russia; Institute of Cytology and Genetics SB RAS, 630089 Novosibirsk, Russia; Agrarian and Technological Institute, Peoples’ Friendship University of Russia (RUDN), 117198 Moscow, Russia; Department of Bioinformatics, College of Life Sciences; First Affiliated Hospital of Medical School, Zhejiang University, Hangzhou 310058, China; Technical Faculty, Bielefeld University, 33501 Bielefeld, Germany

Integrative bioinformatics approaches serve as background for Next-Generation Sequencing-driven analysis for gene regulation studies [1], [2]. This issue of the Journal of Integrative Bioinformatics presents recent work discussed at the BGRS\SB-2020 (Bioinformatics of Genome Regulation and Structure\Systems Biology) conference held in Novosibirsk, Russia (https://bgrssb.icgbio.ru/2020/). Here we collated a number of cancer gene expression studies [3], [4], as well as database development reports [5], [6]. The BGRS conference series on bioinformatics proceeds annually since 1998 under the joint steerage of the Institute of Cytology and Genetics of the Siberian Branch of the Russian Academy of Sciences and Novosibirsk State University [7], [8]. Overall focus of the conference was on the analysis of gene expression regulation in a genome on systems level – such as gene networks and systems biology models [9]. The authors were co-organizers of BGRS meetings and later satellite society meetings on bioinformatics, such as Russian-German Virtual Bioinformatics Network, Sino-Russian workshops on bioinformatics [10] (http://www.zjbioinformatics.org/meetings/2020/index.html; http://glab.hzau.edu.cn/BioInfo/index.html). We had publications in special topic issues after the earlier conferences before in BMC Genetics [11], BMC Medical Genomics, Journal of Bioinformatics and Computational Biology [11], [12], [13] and Frontiers in Genetics [2] and related special journal issues [14] since 2012. The Journal of Integrative Bioinformatics supported this field by organization of special journal issues and promoting international collaboration on bioinformatics [1], [15].

The Systems Biology and Bioinformatics (SBB) School in Novosibirsk (https://conf.icgbio.ru/sbb2020/) were initially conceived as satellite event for young scientists held at the same time as BGRS\SB conference series, becoming now large independent event [11], [12]. In this journal issue we present works by young scientists [3], [4] on database development.

We open this issue with the paper by Sergey Kratov [3] on the Digital Platform “Bioinformatics”. The authors discuss the digital platform in the field of bioinformatics and the formation of the thematically oriented and industrial digital ecosystems. It has broad applications spectrum in human and model organisms’ genetics, agrobiology. Particular attention is devoted to the components of such platform – the project office tool for bioresource collections management (wild and laboratory animals, breeding, museum zoological animal collections, plant collections etc.). This work highlights main approaches and database recourses developed at the Institute of Cytology and Genetics SB RAS such as TRRD (Transcription Regulatory Regions Database) and recent specialized databases.

Next paper by Anton Bogomolov and colleagues [4] presents novel database “Reproductive potential of the male population of Russia” (RPM), which is almost the only source of such information in Russia (www.sysbio.ru/rpm). It was created using the relational database management system MariaDB. The database includes reproductive information of more than a thousand of male volunteers from five large cities of Russia compiling biomedical information.

The following papers highlight genomic studies of particular tumors [5], [6]. Work by Vladimir Kalinin [5] refers to the mathematical models in cancer. The author studied cell interactions in glioma, developing own mathematical model.

Anastasiya Kobelyatskaya et al. [6] discuss prostate cancer study based on DNA methylation. Bioinformatics analysis of omics data allows identifying molecular genetic changes associated with the disease development, as well as markers of prognosis and response to therapy. Alterations in DNA methylation and histone modification profiles widely occur in malignant tumors [15], [16]. In this study, Kobelyatskaya and co-authors analyzed changes in DNA methylation in three groups of prostate cancer patients. CpG sites from several human genes were shown as potential prognostic markers of the high-risk group of prostate cancer. Recently the same authors’ group has discussed the role of miRNA expression signature in the same cancer type [17]. These results also were discussed at BGRS conference series and published in special post-conference journal issues [18].

We have additional research materials on medical bioinformatics and plant genomics that missed this BGRS-2020 special journal issue. Related manuscripts will be submitted to the Journal of Integrative Bioinformatics to be published in the next regular issue(s). New bioinformatics challenges are arising in the context of the pandemic, one example is the analysis of comorbidity on COVID-19 and non-infectious diseases. Such problems were discussed recently at SIBS-2020 – Sechenov International Biomedical Summit in Moscow (https://sechenov-sibs.confreg.org/).

The monitoring of the origin of new virus mutations is of great importance as every new strain may have unique features of interaction with the host organism. The problems of hazards control in food safety related to infectious diseases became important due to the SARS-CoV-2 pandemic [19]. The methods to study association between genes and diseases by bioinformatics tools and literature mining became of great importance [20].

To conclude, this issue includes reports of recent medical genomics applications in cancer and databases development. We aim to support the collaboration in the frames of the BGRS conference and Systems Biology and Bioinformatics Schools by continuing international exchange and education programs for young scientists.
